# Autonomous driving system for single hydrostatic transmission crawler tractor based on multi-modal steering control

**DOI:** 10.3389/fpls.2026.1887292

**Published:** 2026-07-24

**Authors:** Lin Wang, Weiqiang Fu, Hao Wang, Zhijun Meng, Yibo Wei, Weixian Qin, Maochen Zuo, Tielong Yu, Qingshan Meng, Yaxin Ren

**Affiliations:** 1 Xi’an University of Technology, Xi’an, China; 2 State Key Laboratory of Intelligent Agricultural Power Equipment, Luoyang, China; 3 Luoyang Tractor Research Institute Co., Ltd., Luoyang, China; 4 Information Technology Research Center, Beijing Academy of Agriculture and Forestry Sciences, Beijing, China; 5 Beidahuang Group Heilongjiang Qixing Farm Co., Ltd, Jiamusi, China

**Keywords:** autonomous driving, crawler tractor, hydrostatic transmission, robot tractor, state feedback control

## Abstract

**Introduction:**

Autonomous navigation of crawler tractors is essential for precision agriculture; however, existing systems often face a trade-off between path-tracking accuracy and steering stability, particularly under complex field conditions. To address this limitation, this study aimed to develop an autonomous driving system for single-HST (Hydrostatic Transmission) crawler tractors that enhances both control resolution and operational reliability.

**Methods:**

A kinematic model of the single-HST crawler chassis was established, and a state-feedback path-tracking controller integrating lateral and heading deviations was proposed. A pulse-width modulation (PWM)-based multi-modal steering control strategy was designed to enable intelligent switching and smooth transition between differential steering and unilateral braking steering by dynamically adjusting the steering hydraulic cylinder stroke. A three-layer hardware and software architecture—comprising perception, decision-making, and execution layers—was constructed, and an embedded vehicle controller integrating path planning and real-time control was developed. Field tests were conducted at the China National Precision Agriculture Research Demonstration Base, including fixed-curvature path tracking and reciprocating autonomous operation trials.

**Results:**

Under curve path-tracking conditions, the proposed multi-modal steering control achieved an average lateral deviation of 4.25 cm, with a standard deviation below 5.0 cm, representing a 34.4% reduction compared with unilateral braking steering. In reciprocating operations, the average inter-row spacing error was 6.04 cm, satisfying the precision requirements for agricultural machinery under both field soil and cement pavement conditions.

**Discussion:**

The results demonstrate that the proposed system effectively balances tracking accuracy and steering stability in autonomous crawler tractor navigation. The multi-modal steering strategy offers a practical solution for agricultural machinery operating on varied surfaces and shows strong potential for broader application in precision farming systems with similar terrain conditions.

## 1 Introduction

Cultivated land in hilly and mountainous areas accounts for approximately one third of the total cultivated land in China. Owing to complex terrain and fragmented plots, conventional medium- and large-sized agricultural machinery often shows poor adaptability and low operating efficiency, which seriously restricts the modernization of regional agriculture (Yang et al., 2023). Crawler tractors have become an ideal choice for field operations under complex terrain conditions in hilly and mountainous areas because of their low ground pressure, high traction capability, and superior off-road mobility (Takai et al., 2011; Zeng et al., 2026). Among them, the transmission scheme combining a single-path hydrostatic transmission (HST) and a mechanical gearbox has been widely used in small- and medium-sized crawler tractors because of its simple structure, low cost, and convenient maintenance (Wei et al., 2024). However, this type of chassis commonly adopts unilateral braking steering through the coordinated action of steering clutches and brakes. This steering mode has inherent limitations, including poor steering smoothness, low control resolution, severe soil disturbance, and intensified track wear, making it difficult to meet the requirements of high-precision autonomous navigation operations (Zhang et al., 2022).

In recent years, with the rapid development of autonomous agricultural machinery systems, agricultural robots are gradually evolving toward closed-loop perception–decision–execution architectures. These systems typically integrate multi-sensor perception, decision-making, and motion control to enable reliable autonomous navigation in complex field environments. Representative studies have demonstrated that such system-level frameworks significantly improve robustness and field adaptability under real agricultural conditions (Zhao et al., 2025, 2026). Moreover, recent research further emphasizes the importance of real-world deployment and field validation in agricultural robotics, highlighting the gap between algorithm development and practical agricultural applications (Yang et al., 2025).

Extensive studies have been carried out on autonomous navigation and path tracking control of agricultural vehicles and crawler vehicles (Chen et al., 2025; Liu et al., 2026). Takai et al. (2014) from Noguchi’s research group established a relationship model between the yaw rate of a crawler-type robot tractor and the input voltage of an electromagnetic proportional valve through experimental calibration, thereby realizing differential steering control. Path tracking control for brake-steering tracked vehicles has also been investigated based on improved pure pursuit algorithms (Hu et al., 2024). Sabiha et al. (2022) proposed a combined control strategy based on optimized backstepping and sliding mode control. In addition, adaptive look-ahead-distance methods have been developed to improve path tracking performance of crawler tractors (Song et al., 2025). Although high tracking accuracy was achieved, the algorithm was relatively complex and imposed stringent requirements on controller computing capability. In domestic studies, Hu et al. (2026) presented a sensor fusion algorithm effectively enhancing the performance of GNSS/SINS integrated navigation for tractors in low-dynamic environments, meeting the requirements for autonomous navigation systems. Wang and Noguchi (2021) provided a real-time states estimation method for robot tractor navigation using dynamic mode decomposition.

Despite these advances, most existing studies still focus on either path tracking control or single steering mode optimization. Classical geometric methods such as Pure Pursuit and Stanley controllers are widely used due to their simplicity, but they suffer from reduced performance under large curvature and high deviation conditions (Wang et al., 2024). Model-based methods such as sliding mode control and backstepping improve robustness but introduce higher computational complexity. For tracked vehicles, brake-based steering remains dominant; however, it leads to discontinuous motion, increased track wear, and poor steering smoothness (Li et al., 2025).

It should be noted that existing studies on automatic steering of single-HST crawler chassis mainly focus on the optimization of a single steering mode, while collaborative control between differential steering and unilateral braking steering has received insufficient attention (Wang and Noguchi, 2018). For precision operations in complex farmland environments, a single steering mode cannot simultaneously satisfy the multiple requirements of tracking accuracy, operating smoothness, and soil protection.

To address the above problems, this study proposes a multi-modal steering control strategy. The core innovations are as follows: (1) the traditional single steering mode is extended to a multi-modal control system containing five discrete modes, namely straight holding, pure differential steering, light hybrid braking steering, medium hybrid braking steering, and pure braking steering; (2) based on a state feedback path-tracking method, lateral error and heading error are fused and converted into a steering angle, thereby realizing unified quantification of the tracking deviation; and (3) based on the PWM modulation principle, the execution sequence of different modes within one control period is dynamically planned, realizing intelligent switching and smooth transition between differential steering and unilateral braking steering. The proposed strategy retains the high-resolution advantage of differential steering while exploiting the rapid correction capability of unilateral braking steering, achieving a balance among control accuracy, operating smoothness, and soil protection. It provides a systematic solution for autonomous driving of single-HST crawler tractors in hilly and mountainous areas.

## 2 Working principle of the steering system of the single-HST crawler chassis

### 2.1 Structure of the electro-hydraulic steering system of the single-HST crawler chassis

**Figure 1:**
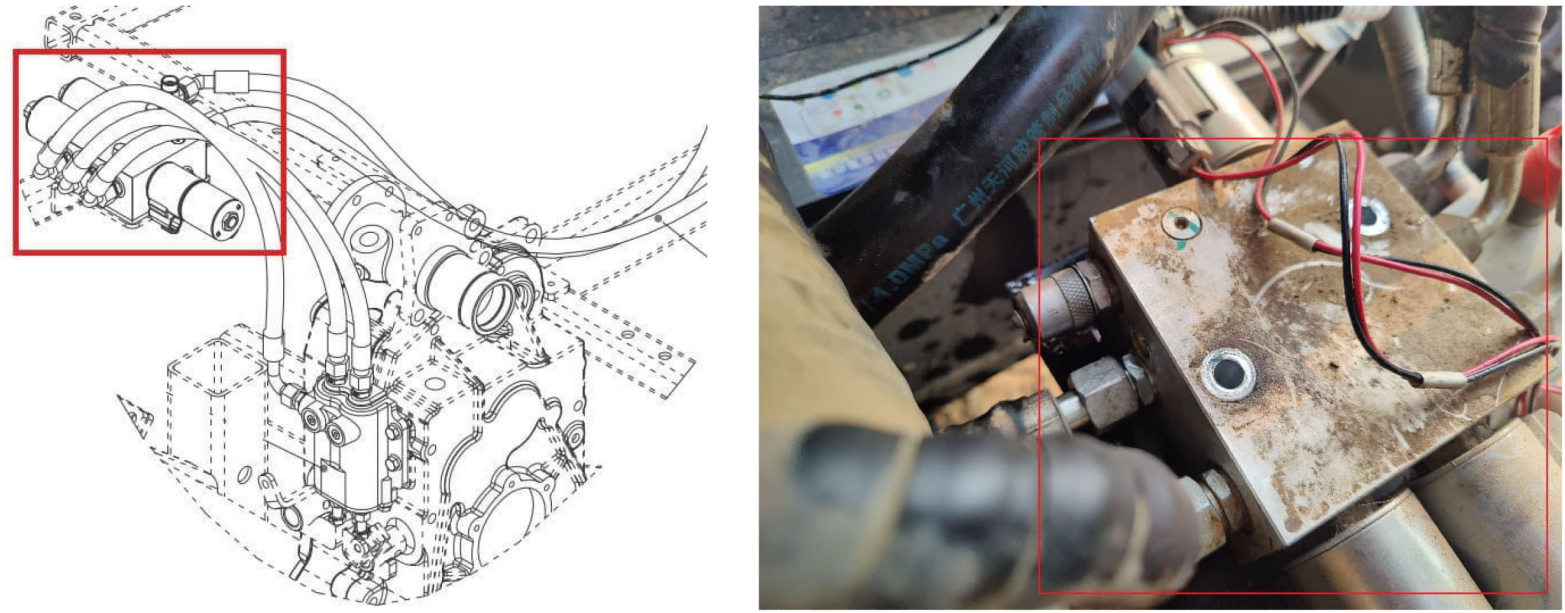
Transmission mechanism and steering actuator layout of the single-HST crawler chassis.

The single-HST crawler chassis adopts a hybrid transmission scheme consisting of a variable pump, a fixed-displacement motor, and a mechanical gearbox. This architecture retains the high efficiency of mechanical transmission while enabling coordinated control of continuously variable speed regulation and differential steering. As shown in **Figure 1**, engine power is transmitted through a belt drive to a Liyuan LY-HPVMF-28-L variable pump. The displacement of the pump is adjusted by changing the swash plate angle through an electric push rod. The pressure oil output by the pump drives a Weizhou YZ60B-CP047 fixed-displacement motor. The motor output shaft is directly connected to a YZ60B gearbox. After internal gear reduction, power is transmitted through a dog clutch to the left and right half shafts and finally drives the tracks.

The mechanical coupling between the steering actuator and the travel transmission chain is a key feature of this chassis. The steering hydraulic cylinder simultaneously controls clutch disengagement and brake engagement through a rocker-arm mechanism. As the piston displacement of the hydraulic cylinder changes, the action sequence of the clutch and brake is guaranteed by the mechanical structure; that is, the clutch is disengaged first and the brake is then applied. This design determines that the switching between differential steering and braking steering depends on precise control of the hydraulic-cylinder stroke rather than two independent actuating mechanisms.

**Figure 2:**
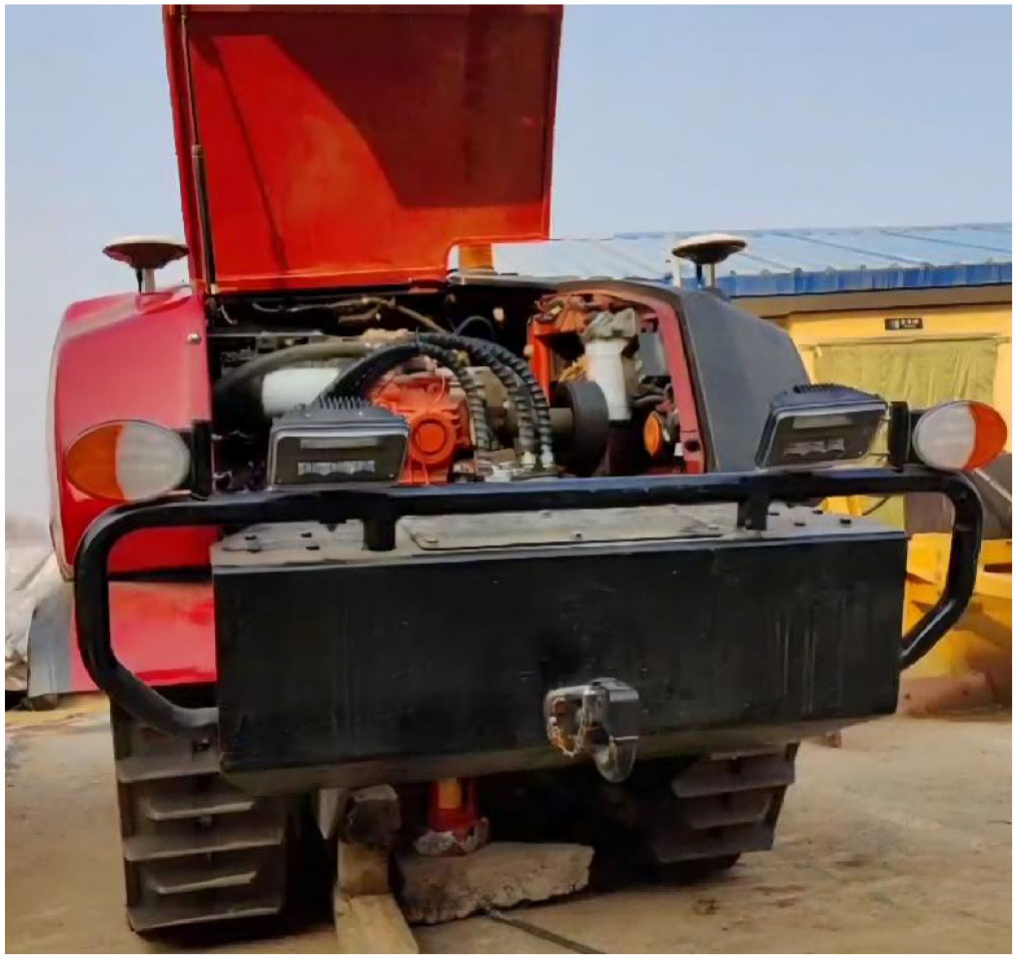
Calibration of the differential steering operating state.

To realize differential steering control of the single-HST crawler chassis, the critical state in which the braking torque is zero was selected as the differential steering operating point. Accurate calibration of this state is the basis of the multi-modal control strategy. The calibration method is shown in **Figure 2**. The chassis was lifted with jacks to eliminate the influence of ground load. At three typical operating speeds of 2, 3, and 5 km/h, the proportional valve duty-cycle signals were tested when the left and right tracks changed from rotating to stopped and from stopped to rotating, respectively.

For the rotating-to-stopped calibration, the duty cycle of the pressure proportional valve was gradually increased from 0% while the chassis was running until the corresponding track stopped rotating; the PWM threshold was then recorded. For the stopped-to-rotating calibration, the duty cycle was gradually decreased from 100% until the track began to rotate. The test results show that, owing to asymmetry caused by mechanical clearance and friction characteristics, the rotating-to-stopped threshold was significantly higher than the stopped-to-rotating threshold in **Table 1**. This hysteresis characteristic should be compensated in the controller program. During path-tracking control, the rotating-to-stopped threshold was selected as the upper limit of differential steering to ensure complete release of the brake and avoid frictional heat generation and power loss.

**Table 1:** Calibration results of the differential state.

Speed (km/h)	Right track proportional valve duty cycle (%) - rotating to stopped	Right track proportional valve duty cycle (%) - stopped to rotating	Left track proportional valve duty cycle (%) - rotating to stopped	Left track proportional valve duty cycle (%) - stopped to rotating
2	36	20	38	26
3	37	20	38	24
5	38	22	38	20

### 2.2 Design of the electro-hydraulic control system

**Figure 3:**
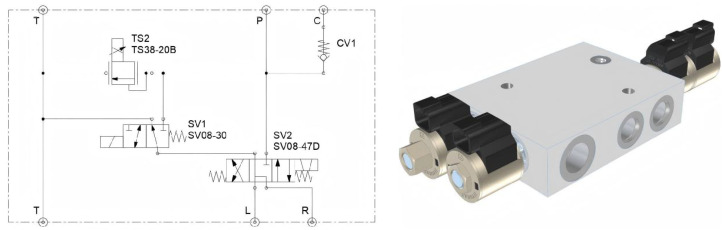
Three-dimensional integrated structure of the steering hydraulic valve group.

The steering electro-hydraulic valve group adopts an integrated HydraForce screw-in cartridge valve scheme. This design is compact and provides reliable sealing. As shown in **Figure 3**, the three-dimensional assembly of the valve group consists of a three-position four-way directional valve (SV08-47D), a two-position three-way directional valve (SV08-30), and a proportional relief valve (TS38-20B), which are screwed directly into the integrated manifold block to form a pipe-free connection. This structure effectively avoids leakage and vibration problems associated with conventional pipe connections. The technical parameters of each component are listed in **Table 2**.

**Table 2:** Parameters of the steering solenoid valve group.

Model	Name	Flow (L/min)	Hysteresis (bar)	Rated current (mA, 12 V coil)	Maximum opening/working pressure (bar)	Pressure drop (bar)	Flow-pressure gradient (L/min/bar)
SV08-47D	Three-position four-way directional valve	11.4	-	1200	207	4.1	-
SV08-30	Two-position three-way directional valve	14	-	1200	207	8.6	-
TS38-20B	Proportional electric relief valve	-	4.55	1100	241	-	0.5

**Figure 4:**
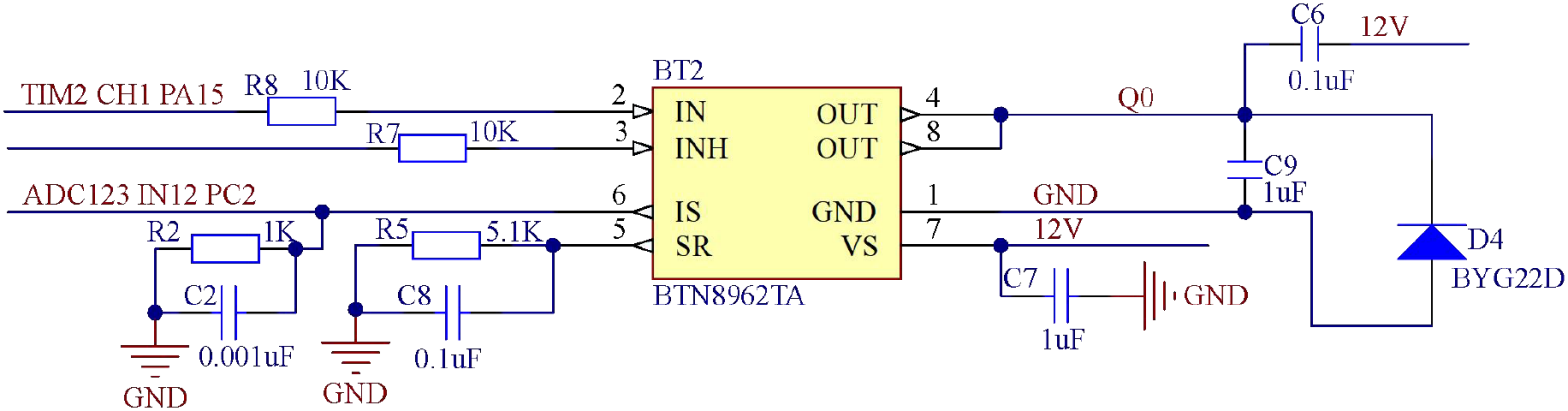
PWM driver circuit for the proportional valve.

The onboard controller must output high-precision PWM signals to drive the proportional valve, and the design of the power output circuit directly affects steering smoothness. As shown in **Figure 4**, an Infineon BTN8962TA half-bridge driver chip was adopted, which integrates current detection and overcurrent protection functions. The PWM signal generated by the MCU is isolated by a TLP2362 optocoupler and then connected to the chip input.

## 3 Design of the autonomous driving system for the single-HST crawler chassis

### 3.1 System architecture

**Figure 5:**
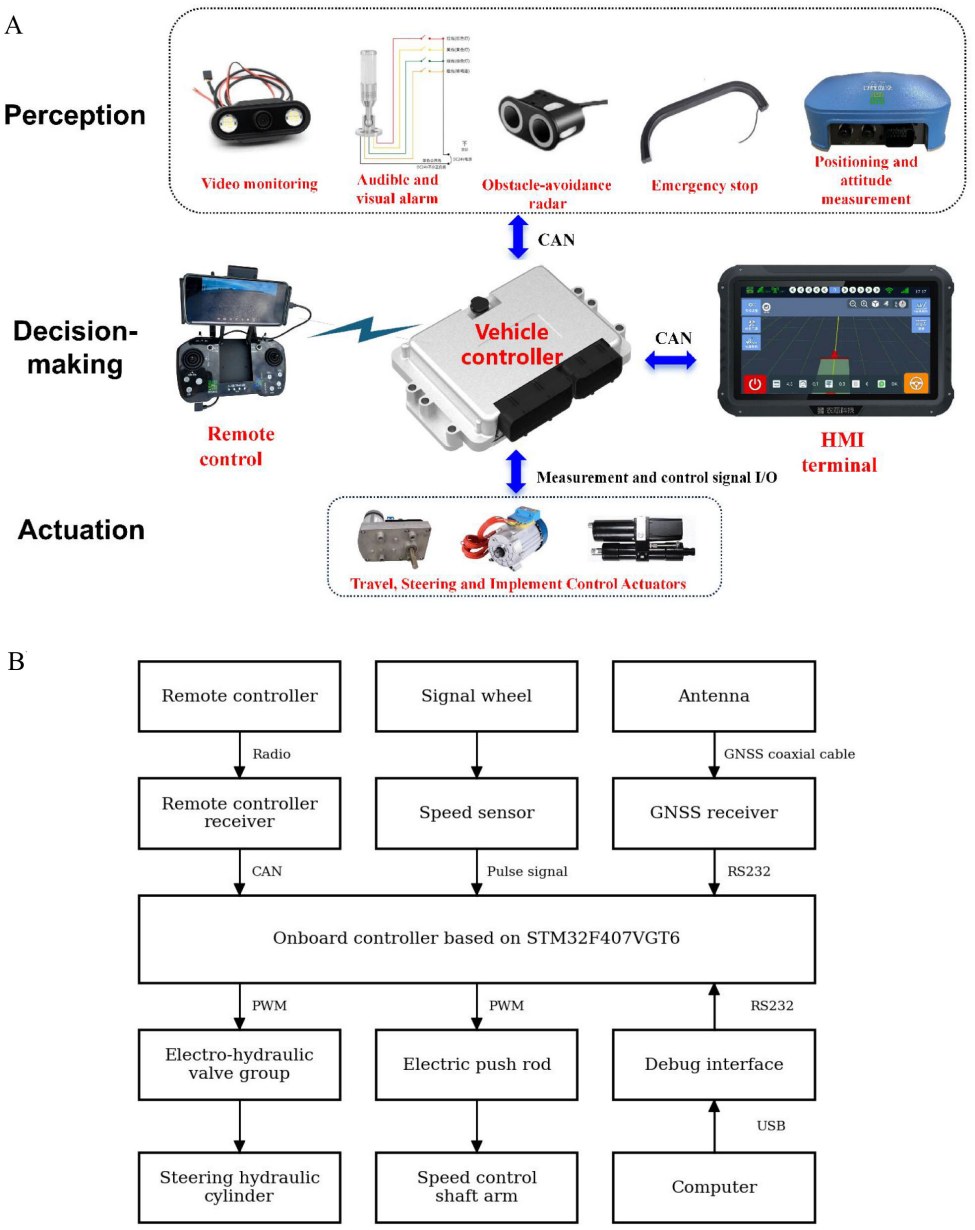
Overall design of the crawler chassis autonomous driving system: **(A)** general L2+ autonomous driving control system architecture; **(B)** overall block diagram of the steering control system.

To meet the requirements of safety, autonomy, and interaction in practical agricultural production, this study proposed a general L2+ autonomous driving control system for agricultural machinery. The system adopts a three-layer architecture, as shown in **Figure 5A**.

1. Perception layer. The perception layer is responsible for collecting environmental and machine-state information and providing basic input data for the control system. It includes multi-level safety devices for environmental perception, such as vision sensors, ranging radar, emergency switches, and audible and visual alarms. It also includes sensors for agricultural machinery and implement operating conditions, including position, attitude, rotational speed, angle, and displacement sensors. Operating-condition sensors are usually installed on actuators. For example, a rotational speed sensor detects changes in the tooth number of a signal wheel rigidly connected to the drive wheel and generates a pulse signal proportional to rotational speed. When the drive wheel rotates, the teeth of the signal wheel pass through the magnetic head of the sensor and generate a square-wave output with a certain frequency. The controller samples this signal through a digital acquisition channel and converts it into the instantaneous linear speed of the drive wheels on both sides of the crawler chassis.
2. Decision layer. The decision layer is responsible for switching between navigation and remote-control modes, managing paths, and executing the steering strategy in real time. It includes the onboard controller, remote controller, and human-machine interaction device. The onboard controller developed based on the STM32F407VGT6 (168 MHz main clock frequency) is the core of the steering control system and is responsible for data acquisition and preprocessing, execution of the differential steering algorithm, execution of the path-tracking algorithm, and output of control signals. The controller operates with a steering update interval of 200 ms. PWM signals used for driving the electro-hydraulic valve group are generated at 500 Hz. The average MCU processing delay is approximately 8.2 ms, which is negligible compared with the system sampling period. GNSS positioning data are received at a frequency of 10 Hz and are fused with wheel-speed signals for real-time navigation control. The remote controller is used to switch the vehicle operating mode. In navigation mode, it records points A and B of the straight line and sets the path. In non-navigation mode, the operator directly controls the motion direction and speed of the crawler chassis through the remote controller for manual driving, path correction, or equipment debugging. Wireless communication is generally used between the remote controller and the onboard controller to ensure stable communication and low latency under complex environments. After entering navigation mode, the controller takes over steering control and executes the differential steering strategy in real time based on GNSS positioning and wheel-speed data. In non-navigation mode, the remote controller can fully control chassis motion and is suitable for turning, row alignment, and recovery from complex terrain conditions.
3. Execution layer. The execution layer receives commands from the onboard controller and completes chassis differential steering, unilateral braking steering, and speed adjustment. It includes the electro-hydraulic valve group, steering hydraulic cylinder, and electric push rod. The electro-hydraulic valve group consists of a proportional electric relief valve, a three-position four-way directional valve, and a two-position three-way directional valve. The proportional electric relief valve adjusts its opening according to the duty-cycle signal and controls the pressure of the steering oil circuit, thereby controlling the working stroke of the steering hydraulic cylinder. The steering hydraulic cylinder drives the steering rocker arm to realize differential steering and unilateral braking steering of the crawler chassis. The electric push rod is mechanically connected to the speed-regulating shaft arm to realize switching among forward travel, reverse travel, and parking.

### 3.2 PWM-based differential steering control method

Assuming that the center of mass and geometric center of the crawler chassis coincide during low-speed operation and that the whole machine is symmetric about its centerline, a two-wheel kinematic model was used to describe the vehicle motion state. Let v_1_ and v_2_ denote the speeds of the left and right tracks, respectively, in m/s, and let b denote the width of the crawler chassis in m. The theoretical steering radius R and steering angular velocity ω of the crawler chassis are expressed as Equations 1, 2.

(1) $$R=\frac{b(v_{1}+v_{2})}{2(v_{2}-v_{1})}$$

(2) $$\omega =\frac{v_{2}-v_{1}}{b}$$

When $v_{1}=v_{2}$, ω=0 and R approaches infinity, indicating that the crawler chassis moves along a straight line. When $v_{1}$ and $v_{2}$ have the same direction, $\frac{\left|v_{2}+v_{1}\right|}{\left|v_{2}-v_{1}\right|}>1,\;R>\frac{b}{2}$, the crawler chassis turns around an instantaneous steering center with a relatively large turning radius. When $v_{1}=0,\;R=\frac{b}{2}$, the linear velocity of the inner track becomes zero, and the crawler chassis rotates around the centerline point of the inner track, corresponding to unilateral braking steering.

If the vehicle speed remains constant, the steering angle of the crawler chassis can be controlled by the duration of unilateral braking. The steering angle is proportional to the action time, and the proportional coefficient is the steering angular velocity. Taking left steering as an example, during unilateral braking steering, the inner track speed $v_{1}$ is zero and the vehicle speed is fixed; therefore, the steering angular velocity reaches its maximum value ω max. During differential steering, $v_{1}$ is not zero and the steering angular velocity is lower than ω max. Thus, under the same action time, differential steering can make the crawler tractor rotate through a smaller angle. Differential steering improves the control resolution of steering angular velocity and helps improve path-tracking accuracy, whereas unilateral braking steering has the advantage of rapid response during headland turning and other large-angle steering operations.

### 3.3 Multi-modal steering control strategy

In this study, steering control was abstracted into five discrete modes, as listed in **Table 3**, forming the core of the multi-modal steering control strategy. Mode 0 is the holding mode and is used to eliminate steady-state oscillation. Mode 1 is the pure differential mode and is suitable for fine correction under small deviations. Modes 2–4 are hybrid modes. By dynamically adjusting the action ratio between T_H_ and T_F_ within one control period T (T = 200 ms), a smooth transition from gentle to aggressive steering can be achieved. This design retains the high-resolution advantage of differential steering while exploiting the rapid correction capability of braking steering.

**Table 3:** Multi-modal steering control logic.

Steering angle range	Mode no.	Steering characteristic	Action sequence				
(0, δ_1_]	0	Straight holding	F	F	F	F	F
(δ_1_, δ_2_]	1	Pure differential steering	T_H_	T_H_	T_H_	T_H_	T_H_
(δ_2_, δ_3_]	2	Light hybrid braking steering	T_F_	F	T_H_	T_H_	T_H_
(δ_3_, δ_4_]	3	Medium hybrid braking steering	T_F_	T_F_	F	T_H_	T_H_
(δ_4_, +180]	4	Pure braking steering	T_F_	T_F_	T_F_	T_F_	T_F_

During path tracking, the lateral deviation d, defined as the perpendicular distance from the vehicle to the target path line, and the heading deviation Δ, defined as the angle between the vehicle heading and the tangent direction of the target path, were used as feedback variables. The controller calculates the required steering angle δ using a state-feedback algorithm, as shown in Equation 3.

(3) δ=Δ+α 

In Equation 3, δ is the correction term for heading error, and its calculation is shown in Equation 4.

(4) $$\alpha =\{\begin{array}{ll} 0\;, & |d|\leq D_{\min}\\ \frac{d}{\left|d\right|}\cdot \frac{\left(|d|-D_{\min}\right)}{D_{\max}-D_{\min}}\cdot \alpha_{\max}, & D_{\min}<|d|\leq D_{\max}\\ \frac{d}{\left|d\right|}\cdot \alpha_{\max}, & |d|>D_{\max} \end{array}$$

Where $D_{\min}$ and $D_{\max}$ denote the minimum and maximum threshold correction distances, respectively; $\alpha_{\max}$ represents the maximum allowable correction angle. Based on the field operational constraints and hardware characteristics of the tracked tractor, these key parameters are configured as: $D_{\text{min}}=0.05\text{m}$, $D_{\max}=1.00\text{m}$, and $\alpha_{\max}=45.0$. Specifically, when the tracked tractor deviates to the right side of the planned path, the lateral deviation is defined as positive (d > 0), which generates a positive correction angle ( α>0) according to (Equation 4) to drive the vehicle to steer leftward toward the baseline. Conversely, when the vehicle is on the left side of the path, the deviation is negative (d < 0), resulting in a negative correction angle ( α<0) that commands a rightward steering adjustment. The sign of the heading deviation Δ follows an identical geometric convention to ensure seamless vector superposition in (Equation 3). In addition, (Equation 3) is selected as a state-feedback–based control structure combining lateral deviation and heading deviation under a small-angle kinematic assumption. This formulation is widely adopted in agricultural vehicle path tracking because it provides a direct mapping from measurable state errors to steering commands while maintaining computational simplicity and real-time feasibility.

Second, the control gains were determined through a combination of simulation-based tuning and field calibration. Specifically, the proportional weighting between lateral deviation and heading deviation was adjusted to achieve a balance between tracking accuracy and steering smoothness. The final parameters were selected to ensure fast convergence without inducing oscillatory steering behavior under varying soil and surface conditions.

## 4 Field test and result analysis

### 4.1 Test platform and experimental scheme

**Figure 6:**
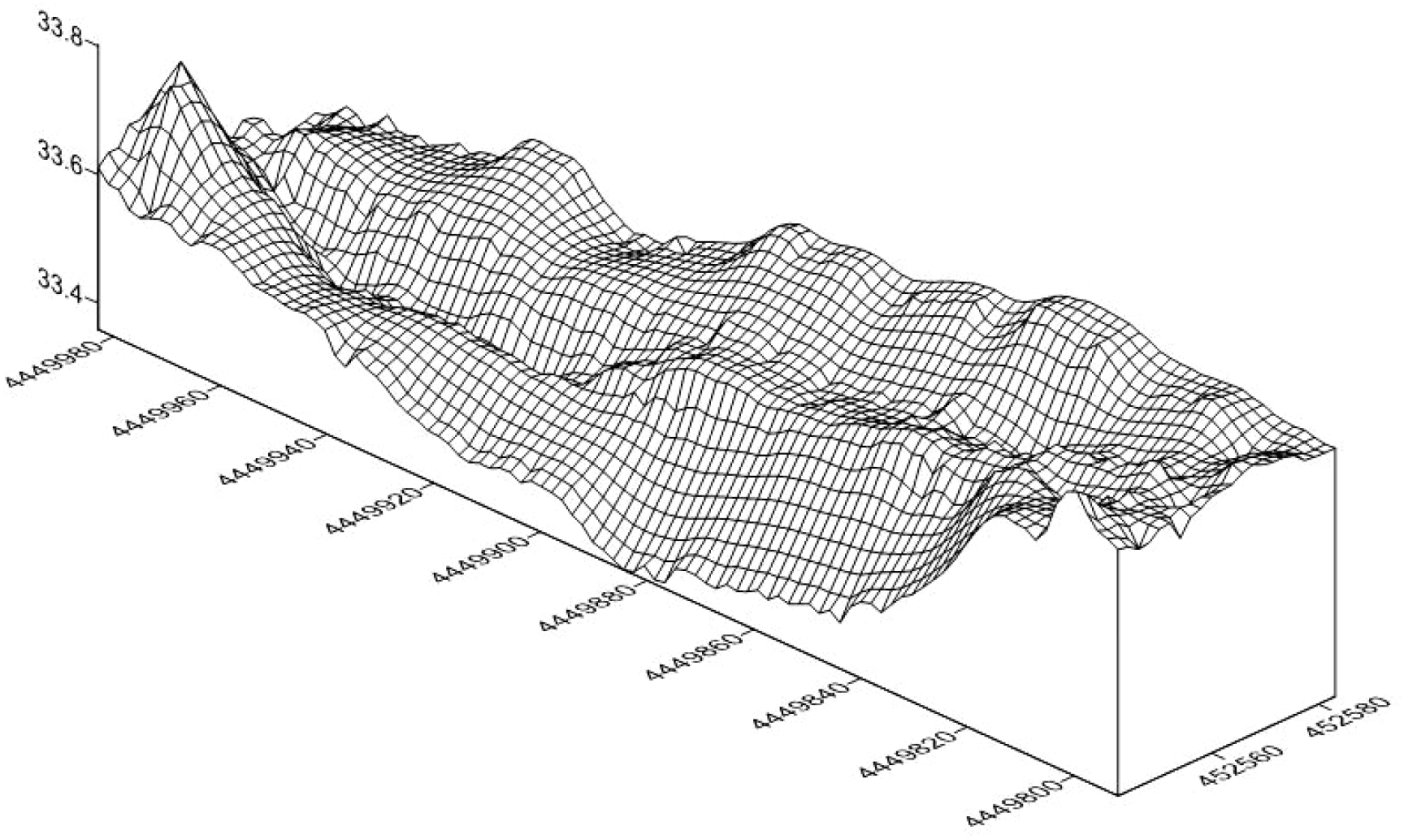
Topographic map of experimental site.

The test platform was a Dongfanghong CR502 crawler tractor, and its main parameters are listed in **Table 4**. Field tests were conducted at the National Precision Agriculture Research Demonstration Base. The topographic map of the experimental site is shown in **Figure 6**.The site selected for this experiment is a conventional field with relatively flat terrain and relatively uniform soil conditions, and the ground elevation difference within the test area is controlled within 20 cm. The tests consisted of two parts: fixed-curvature curve tracking tests in the field, and autonomous adjacent-row tracking tests in the field.

**Table 4:** Main parameters of the Dongfanghong CR502 crawler power chassis.

Parameter	Value
Overall dimensions (L×W×H) (mm × mm × mm)	3100 × 1600 × 1485
Mass (kg)	1800
Chassis width (mm)	1000
Hydraulic system pressure (MPa)	18
Speed (km/h)	0~8.51

### 4.2 Calibration of sensors and actuators

The steering control system built on the Dongfanghong CR502 crawler power chassis regulates vehicle traveling speed through an electric push rod. Under a fixed gear position, the working stroke of the electric push rod determines both the magnitude and direction of vehicle speed. The working stroke of the electric push rod is reflected by the voltage of the push rod potentiometer read by the controller. To achieve vehicle speed control, the relationship between the push rod potentiometer voltage and vehicle speed direction was calibrated. When the vehicle is in the parking state, the average potentiometer voltage is 1.73 V; when the vehicle is in the maximum reverse gear, the average potentiometer voltage is 3.36 V.

**Figure 7:**
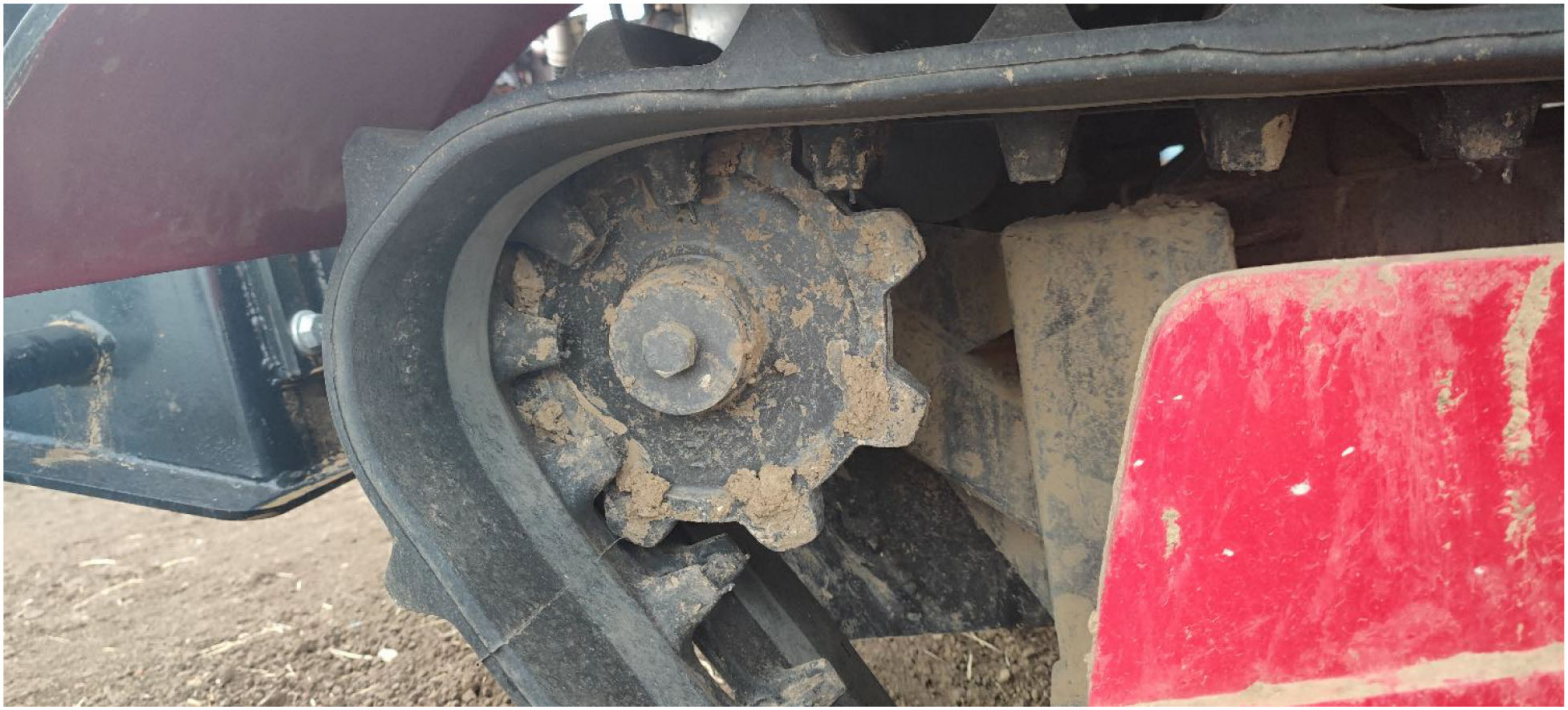
Crawler-type chassis drive wheel.

In crawler vehicle speed measurement, the traditional calculation method based on drive wheel radius has inherent errors. As shown in **Figure 7**, the actual effective engagement radius between the track and drive wheel is affected by multiple factors including track tension, wear condition, and ground conditions, resulting in significant deviation between the theoretical radius and effective engagement radius, which directly affects speed measurement accuracy. To address this issue, this study proposes a pulse calibration method based on actual traveling distance. This method first requires the tested vehicle to travel a known distance along a straight line (100 m in this experiment), precisely recording the total number of pulses output by the speed sensor during this process. Through calculation, the actual traveling distance corresponding to each pulse, namely the pulse equivalent, was obtained; the measured value was 1.55 cm. During actual measurement, by counting the number of pulses per unit time and combining it with the pre-calibrated pulse equivalent, the instantaneous vehicle speed can be directly calculated. The advantage of this method is that it completely avoids the difficulty of measuring the effective engagement radius of the drive wheel while automatically compensating for systematic error factors such as drive wheel wear.

**Figure 8:**
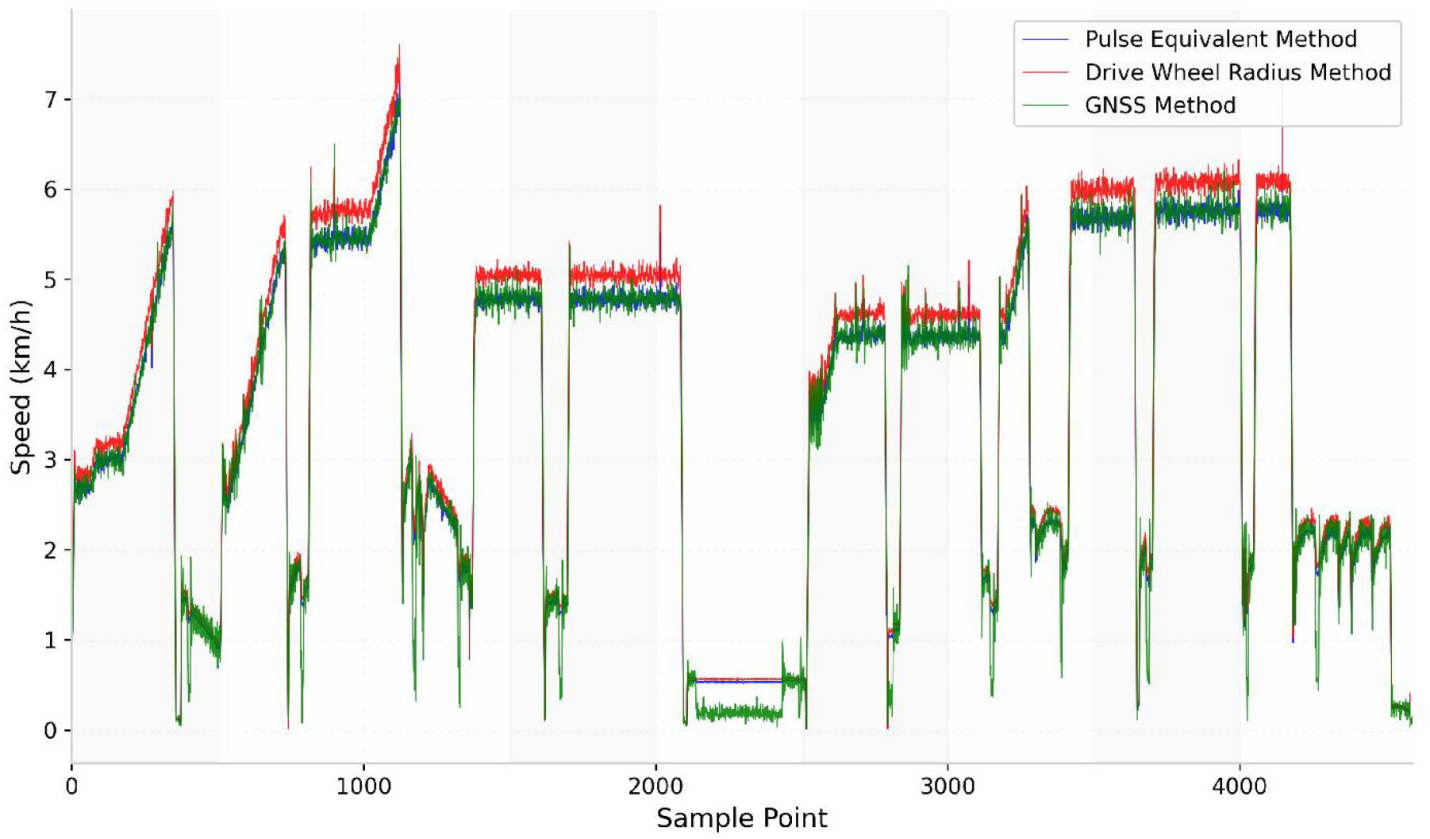
Time-series comparison of speed measurement results from three methods during crawler vehicle operation: pulse equivalent method, drive wheel radius method, and GNSS reference.

**Figure 8** presents the time-series comparison of speed measurement results obtained from three different methods during crawler vehicle operation. It can be observed that the pulse equivalent method (blue curve) closely follows the GNSS reference (green curve) throughout the entire speed range, whereas the drive wheel radius method (red curve) consistently exhibits a positive offset relative to the GNSS measurement. This systematic deviation becomes more pronounced at higher speeds, indicating that the theoretical drive wheel radius overestimates the actual effective radius under operational conditions.

**Figure 9:**
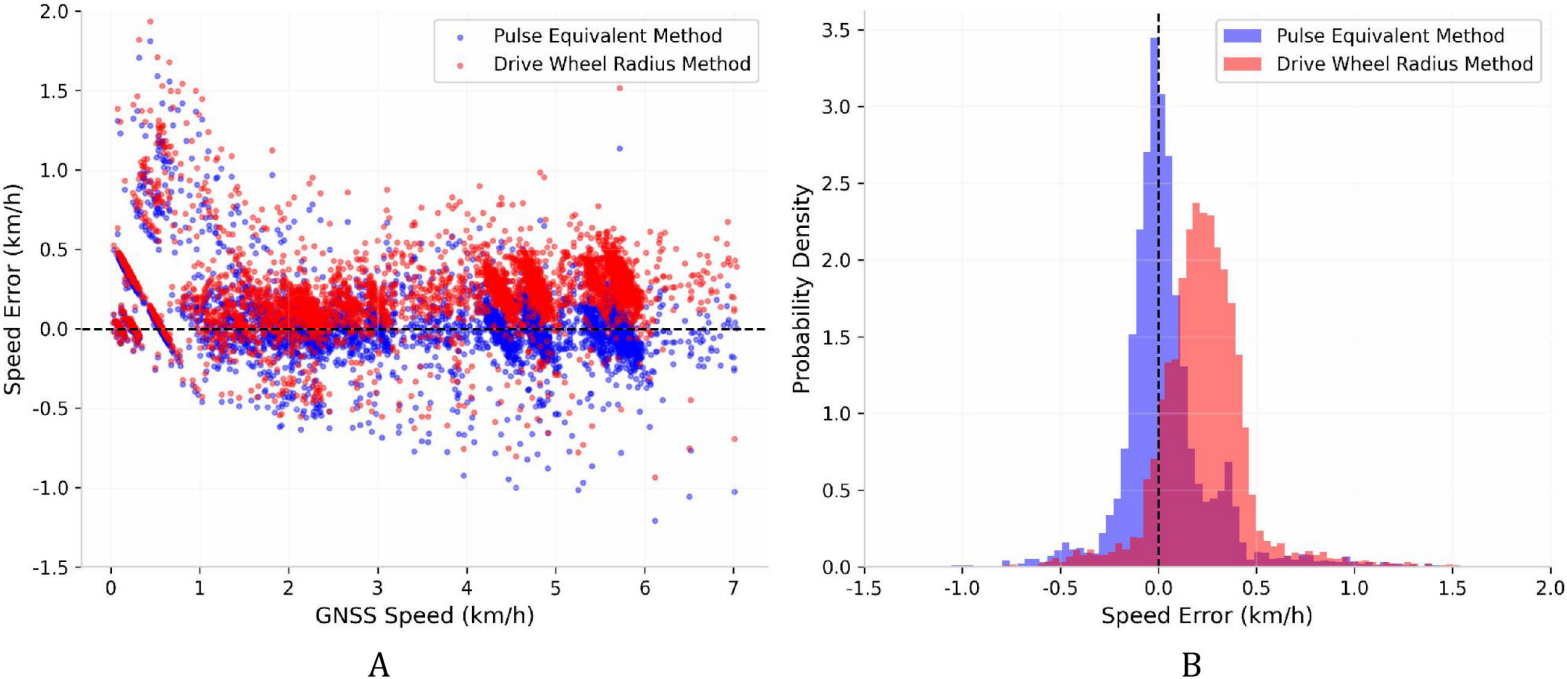
Error analysis of the pulse equivalent method and drive wheel radius method relative to GNSS. **(A)** Error scatter plot as a function of GNSS speed; **(B)** Probability density distribution of speed errors.

To quantitatively evaluate the accuracy of the two calculation methods, the speed errors relative to the GNSS reference were analyzed. As illustrated in **Figure 9**, the error scatter plot (A) reveals that the pulse equivalent method produces errors distributed approximately symmetrically around zero across the entire speed range, with no significant speed-dependent bias. In contrast, the drive wheel radius method exhibits a clear positive systematic error that increases with vehicle speed. The probability density distribution (B) further demonstrates that the error distribution of the pulse equivalent method is centered near zero with a narrow spread, whereas the drive wheel radius method shows a right-shifted distribution, confirming its systematic overestimation tendency.

The statistical results are summarized in **Table 5**. The pulse equivalent method achieves a mean error of 0.037 km/h and an RMSE of 0.248 km/h, both significantly lower than those of the drive wheel radius method (mean error of 0.231 km/h and RMSE of 0.333 km/h). The MAE of the pulse equivalent method is 0.152 km/h, approximately 42.6% lower than that of the drive wheel radius method (0.265 km/h). These results indicate that the pulse equivalent method not only eliminates the systematic error inherent in the drive wheel radius method but also provides higher overall measurement precision, making it more suitable for accurate speed control in crawler vehicles.

**Table 5:** Results of different speed calculation methods.

Method	Mean error (km/h)	Std dev (km/h)	MAE (km/h)	RMSE (km/h)
Pulse Equivalent	0.0372	0.2449	0.1523	0.2477
Drive Wheel Radius	0.2309	0.2397	0.2652	0.3328

### 4.3 Path tracking test

#### 4.3.1 Fixed-curvature curve tracking test

To verify the curve-tracking capability of the steering control system, a fixed-curvature curve tracking test was carried out in the field. The Dongfanghong CR502 crawler power chassis used in the test had a chassis width of 1 m. Considering mounted implement operation, a circular trajectory tracking test with a radius of 3 m was conducted.

Taking circular trajectory tracking with left steering as an example, when the steering radius R is determined and the winding speeds of both tracks can be measured, the theoretical speed of the low-speed-side track can be calculated using Equation 5.

(5) $$v_{1}=\frac{2R-b}{2R+b}v_{2}\;$$

The actual winding speed of the low-speed-side track can be measured by the rotational speed sensor. According to the difference between the measured speed and the theoretical speed, a steering control method similar to that in **Table 3** can be established to control the winding speed of the low-speed-side track so that the turning-radius requirement is satisfied.

**Figure 10:**
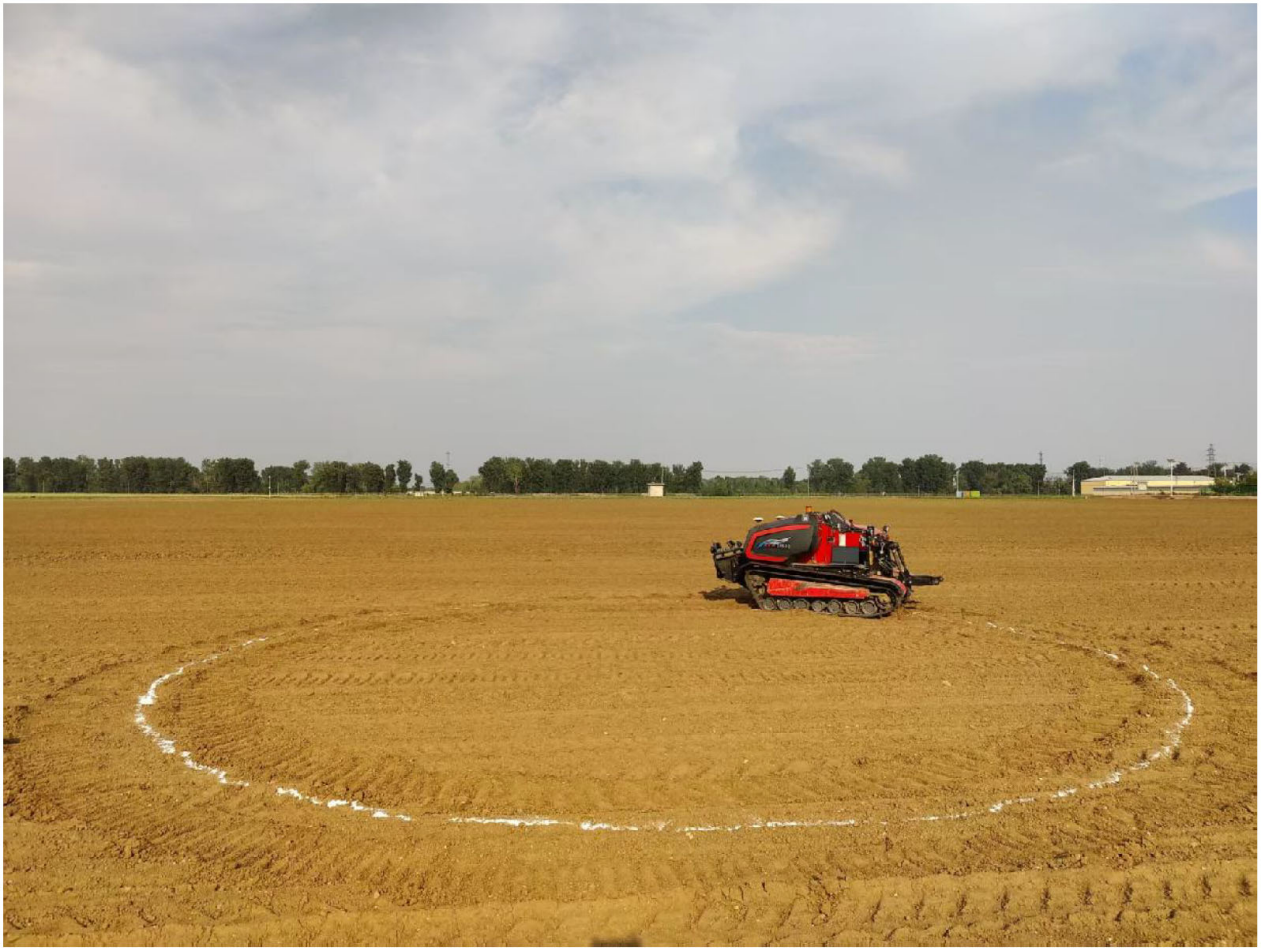
Circular trajectory tracking test.

As shown in **Figure 10**, the crawler chassis was tested on field ground under two steering modes for fixed-curvature curve tracking. The test speed was 2 km/h.

**Figure 11:**
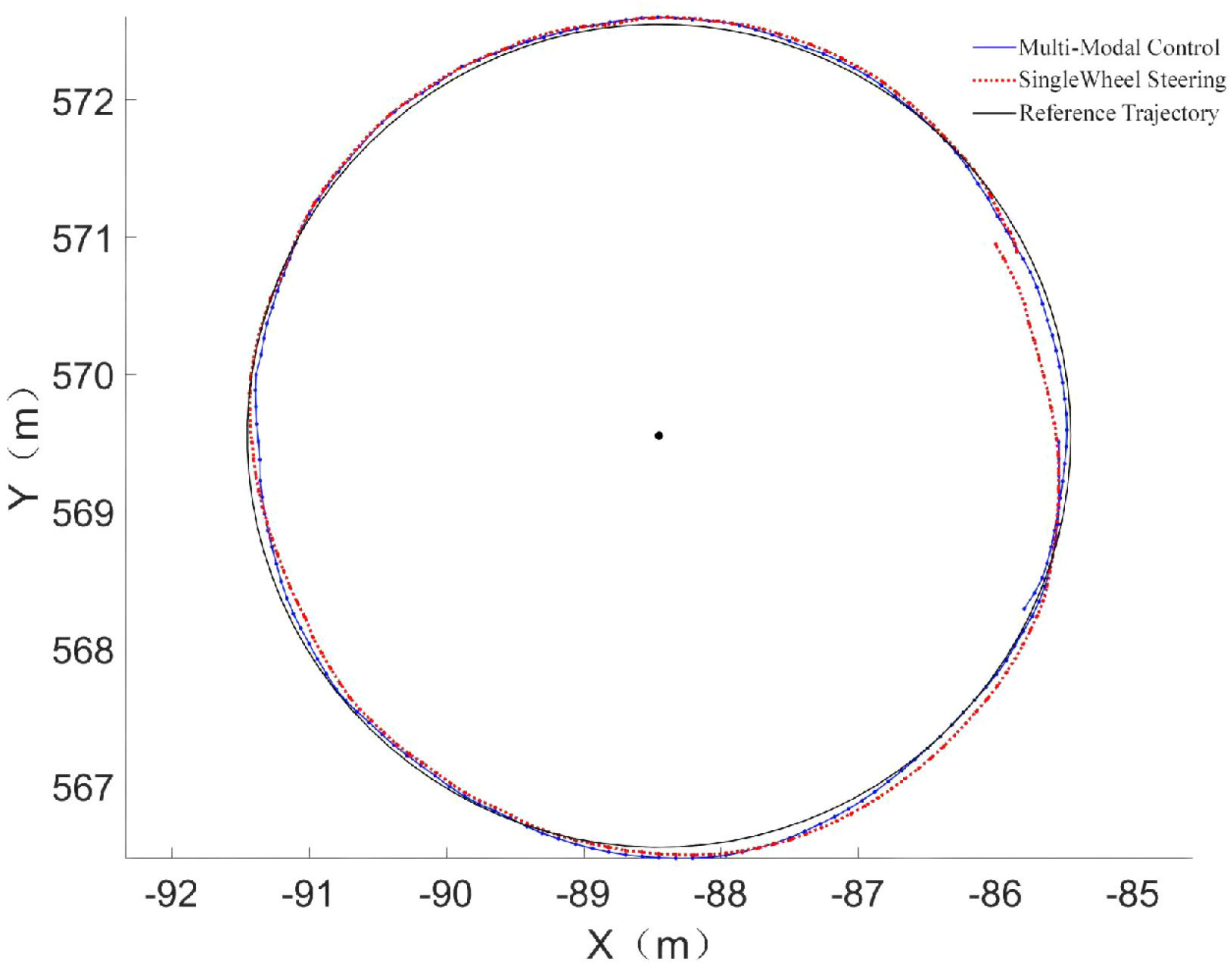
Circular path tracking trajectory.

**Figure 12:**
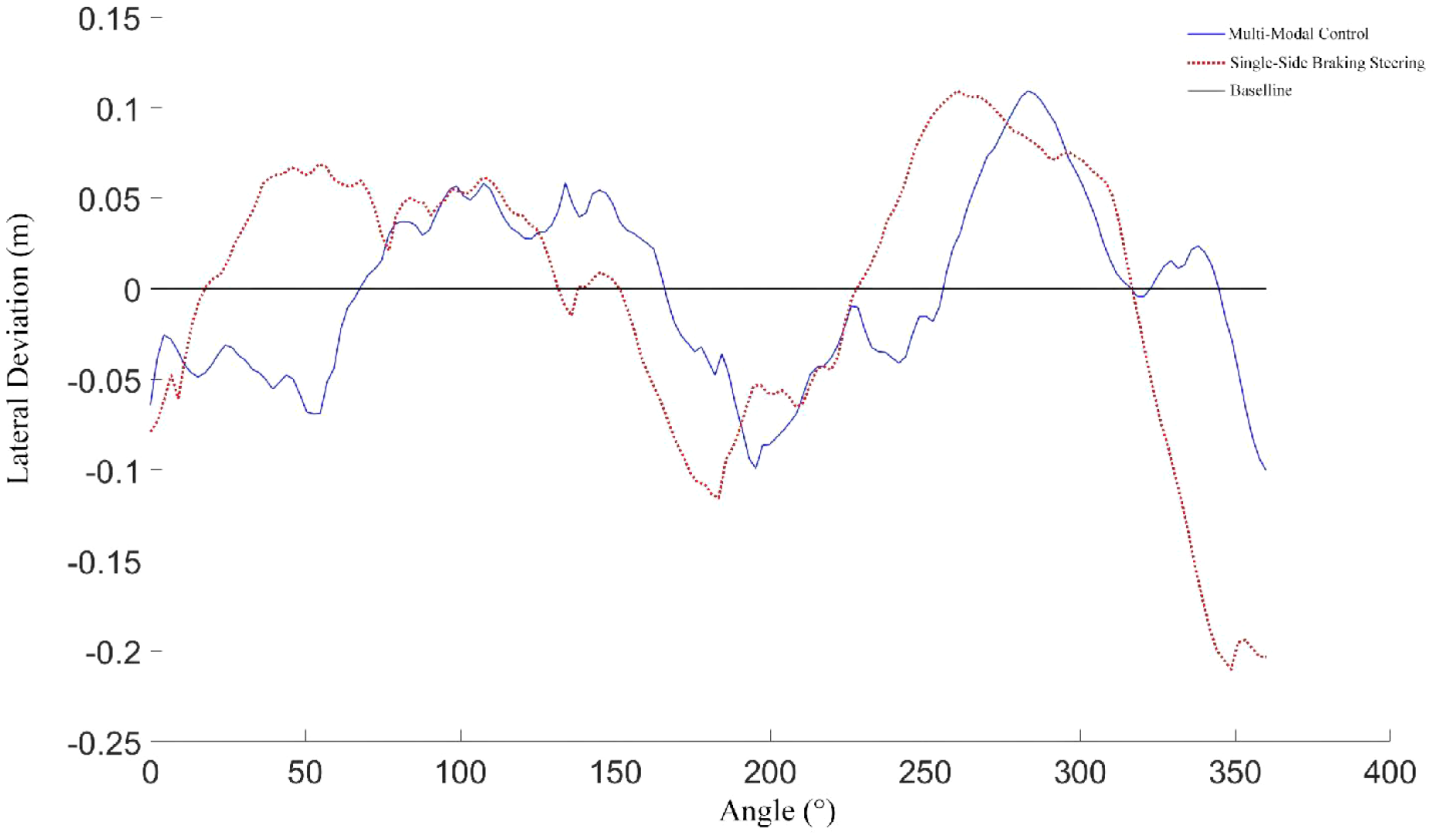
Variation curve of lateral error during circular path tracking.

**Figure 11** records the tracking trajectories of the crawler chassis under the two steering modes. **Figure 12** presents the variation in lateral tracking error. Under multi-modal control strategy, the mean lateral tracking error was 4.25 cm, and the standard deviation was 4.98 cm. In contrast, under unilateral braking steering, the mean lateral tracking error was 6.48 cm, and the standard deviation was 7.96 cm. The experimental results indicate that, in the fixed-curvature circular tracking test, differential steering outperformed the steering method using only unilateral braking steering.

#### 4.3.2 Reciprocating autonomous operation test

**Figure 13:**
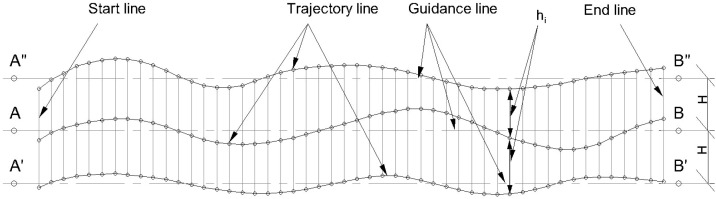
Schematic diagram of the precision test for connecting row spacing.

To verify the autonomous operation capability of the steering control system, a reciprocating operation tracking test was conducted in the field. According to the requirements of the Agricultural Machinery Extension Appraisal Outline (DG/T 157-2023), when the agricultural machine drives toward the navigation line adjacent to the right side of navigation line AB, the assisted driving system should enter a stable working state before reaching the starting line. In assisted driving mode, the machine starts from the starting line at low speed (1.0-2.5 km/h) and medium speed (8.5-9.5 km/h), passes through the terminal line, and records the actual trajectory. As shown in **Figure 13**, the trajectories at the starting line and terminal line were divided into 49 equal intervals, producing 50 intersection points on the trajectory. The connecting row-spacing deviation was obtained by subtracting the preset working trajectory spacing from the row spacing of each pair of intersection points.

The mean error of the connecting row spacing for one operation was calculated using Equation 6.

(6) $$\bar{h}=|\frac{1}{N}\sum_{i=1}^{N}\left(h_{i}-H\right)|$$

In Equation 6, $\bar{\text{h}}$ denotes the average error of connecting row spacing, in centimeters (cm), ${\text{h}}_{\text{i}}$ is the trajectory spacing at the i-th sampling point, in centimeters (cm), H represents the preset connecting row spacing, in centimeters (cm).

The connecting row-spacing accuracy was calculated using Equation 7. ${\text{S}}_{2}$ denotes the precision of connecting row spacing, in centimeters (cm).

(7) $$S_{2}=\sqrt{\frac{1}{N-1}\sum_{i=1}^{N}{\left[(h_{i}-H)-\frac{1}{N}\sum_{i=1}^{N}\left(h_{i}-H\right)\right]}^{2}}\;$$

The test was then repeated on the navigation line adjacent to the left side of navigation line AB. The maximum value of the mean connecting row-spacing error and the maximum value of connecting row-spacing accuracy from the left and right operations were taken as the final measurement results.

For the crawler chassis, on the basis of straight-line tracking, the straight line formed by points A and B was used as the reference path, and parallel straight paths were generated by offsetting it with a fixed working width. When the vehicle reached the boundary point, a row-to-row steering command was triggered. By using the rotational speed sensor as an odometer and RTK-GNSS heading information as feedback, the crawler chassis could complete the sequence of straight-line tracking, steering, traveling over a fixed working width, steering again, and tracking the next straight line, thereby realizing fixed-width adjacent-row operation. In this adjacent-row tracking test, the speed was set to 2 km/h, and the working width was set to 1.2 m.

**Figure 14:**
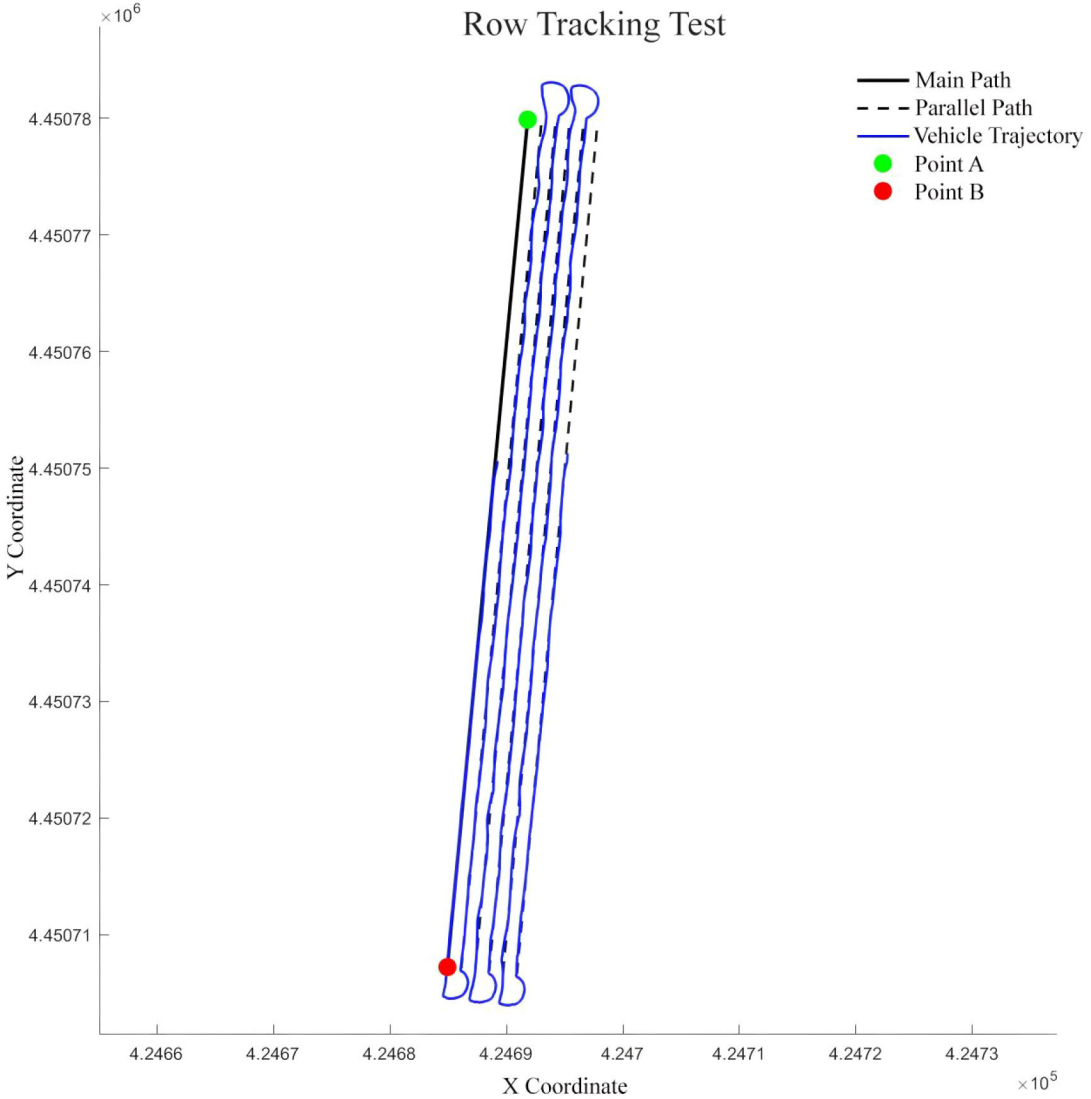
Adjacent-row tracking trajectory.

**Figure 15:**
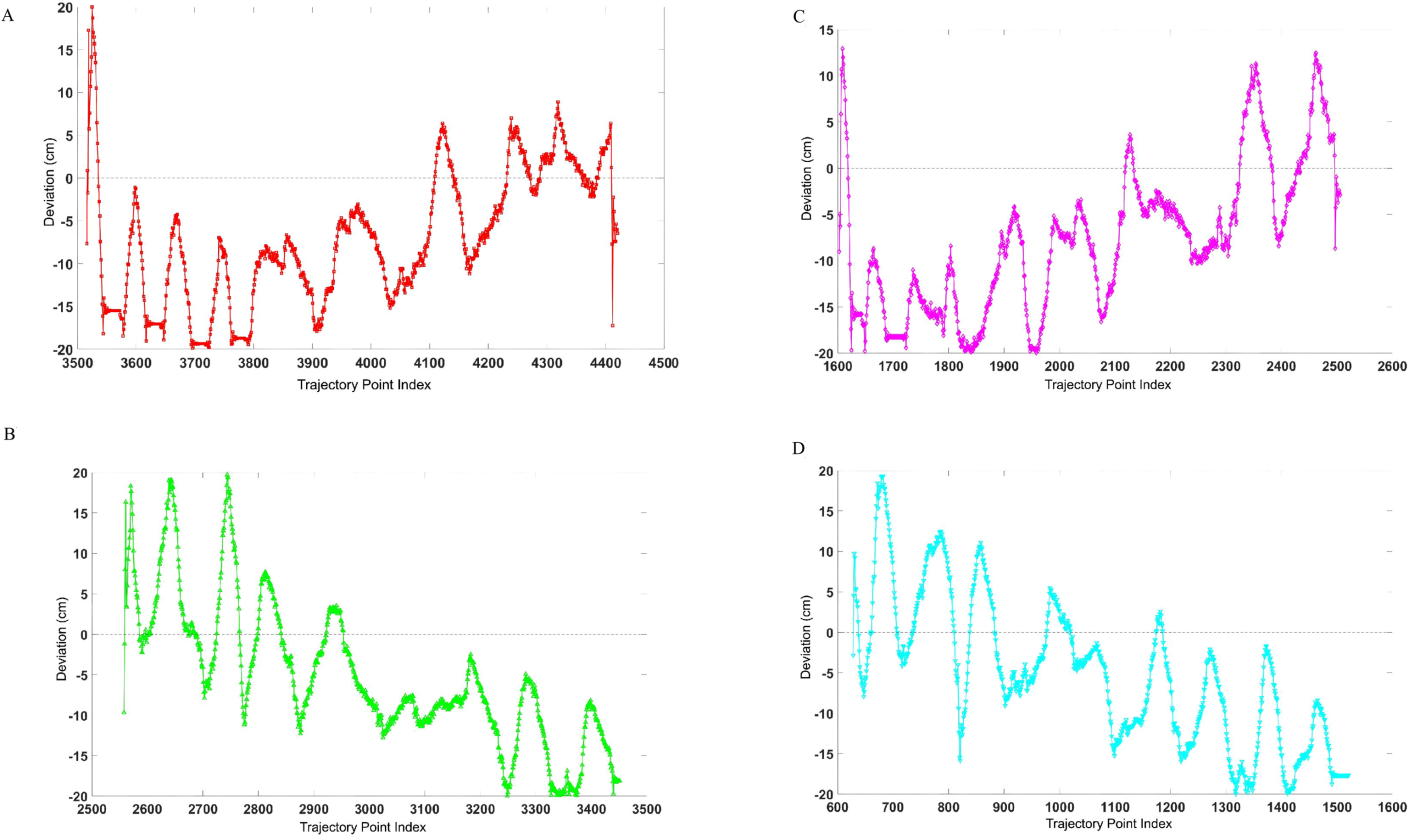
Lateral error in adjacent-row tracking: **(A)** path 1; **(B)** path 2; **(C)** path 3; **(D)** path 4.

**Figure 14** shows the tracking trajectory of the crawler chassis during the adjacent-row tracking test in the field. **Figure 15**. Lateral error in adjacent-row tracking: (A) path 1; (B) path 2; (C) path 3; (D) path 4. **Table 5**. Results of different speed calculation methods. Records the variation in lateral tracking error for each adjacent path after steering. **Table 6** gives the mean absolute lateral deviation and standard deviation for each adjacent path, as well as the mean connecting row-spacing error and connecting row-spacing accuracy calculated according to the equations.

**Table 6:** Results of the adjacent-row tracking test.

Adjacent path	Mean lateral deviation (cm)	Standard deviation of lateral deviation (cm)
1	8.93	5.64
2	8.67	5.38
3	9.61	5.48
4	8.72	5.58
$\bar{h}=6.04\text{cm}$	S_2_ = 5.32cm	

## 5 Conclusions and future work

To meet the requirements of autonomous navigation operation of single-HST crawler tractors in hilly and mountainous areas, this study proposed and verified a multi-modal steering control system. By integrating the advantages of differential steering and unilateral braking steering, the system effectively addresses the contradiction among steering smoothness, control accuracy, and soil protection in the conventional unilateral braking steering mode. The experimental results showed that the proposed multi-modal control strategy achieved a mean lateral error of 4.25 cm during curve path tracking, reducing the average lateral deviation by 34.4% compared with the unilateral braking steering mode. The connecting row-spacing error during reciprocating operation was 6.04 cm. The embedded controller architecture and PWM modulation method adopted in the system provide a feasible solution for low-cost and highly reliable autonomous driving of agricultural machinery.

Although the expected results were achieved, several aspects still require further improvement. The current tests were mainly conducted on cultivated land and cement pavement, and verification under more complex typical hilly and mountainous conditions, such as paddy fields and steep slopes, remains insufficient. In addition, path planning in the system is currently limited to simple straight paths and fixed-curvature paths, and the tracking capability for obstacle avoidance and variable-curvature headland turning paths requires further study. The switching thresholds of the multi-modal steering strategy were determined through experimental calibration, and an adaptive adjustment mechanism has not yet been established. During steering and turning operations, track slip and skid were relatively significant, affecting the accuracy of large-angle steering. However, direct soil-related indicators such as soil compaction, slip ratio, disturbed area, energy consumption, and hydraulic pressure fluctuation were not measured in this study, which limits the quantitative evaluation of soil disturbance reduction. This will be addressed in future work. It should be noted that the experiments in this study were conducted under cement pavement and field conditions. Although these environments can represent typical operating surfaces for agricultural machinery, more complex terrains such as steep slopes, muddy soil, and highly uneven surfaces were not included in the current experimental validation. Therefore, the applicability of the proposed method to highly rugged mountainous environments should be interpreted with caution and requires further verification in future work. These limitations restrict the adaptability and intelligence of the system under more complex environments.

Future research will focus on three aspects. First, the experimental scenarios will be expanded to verify system stability on more ground types, such as paddy fields and sloped land. Second, global path-planning algorithms will be developed by combining terrain information and operation-task requirements to improve operating efficiency. Third, advanced methods such as deep learning and reinforcement learning will be explored to realize intelligent switching and adaptive control of steering modes, further improving system adaptability and reliability under complex operating conditions and providing stronger technical support for agricultural mechanization in hilly and mountainous areas.

## Data Availability

The raw data supporting the conclusions of this article will be made available by the authors, without undue reservation.
